# Successful ageing is associated with falls among older adults in India: a large population based across-sectional study based on LASI

**DOI:** 10.1186/s12889-024-19181-7

**Published:** 2024-06-24

**Authors:** Yujing Wang, Siqi Leng, Yuming Jin, Xiangdong Tang, Xian Zhu, Lina An

**Affiliations:** 1 Department of Emergency and Critical Care Medicine, Shanghai General Hospital, Shanghai Jiao Tong University School of Medicine, Shanghai, China; 2 Sleep Medicine Center, Mental Health Center, Department of Respiratory and Critical Care Medicine, West China Hospital, Sichuan University, Dian Xin Nan Jie 28#, Chengdu, 610041 China; 3 Department of Urology, Institute of Urology and National clinical Research Center for Geriatrics, West China Hospital of Sichuan University, Chengdu, China; 4 Department of Geriatrics, Shanghai General Hospital, Shanghai Jiao Tong University School of Medicine, Shanghai, China

**Keywords:** Falls, Successful ageing, Age

## Abstract

**Background:**

Falls are common in the elderly and can lead to adverse consequences, like injuries, hospitalization, disability even mortality. Successful ageing emerged in sight to assess physical, psychological and social status of older adults. This study is conducted to explore the association between them in a large Indian community-dwelling population.

**Methods:**

Data were based on the wave 1 survey of the Longitudinal Ageing Study in India (LASI). People aged 60 and above with complete information were included. The elderly met five standards including absence of chronic diseases, freedom from disability, high cognitive ability, free from depressive symptoms and active social engagement, were classified into successful agers. The assessment of falls, fall-related injuries and multiple falls depended on interview. Multivariate logistic regression was conducted to find the associations between falls, fall-injury, multiple falls and successful ageing after adjusting both socio-demographic and biological covariates. The log-likelihood ratio test was calculated interactions in subgroups.

**Results:**

31,345 participants in LASI were finally included in our study. Of them, 20.25% reported fall, and 25% were classified into successful agers. After full adjustment, successful ageing was negatively associated with falls (OR 0.70; 95%CI 0.65–0.76) and multiple falls (OR 0.70; 95%CI 0.63–0.78). And the association did not show the significance in older adults with fall-related injuries (OR 0.86; 95%CI 0.72–1.04).

**Conclusions:**

Successful ageing was negatively associated with falls and multiple falls, but not fall-related injuries in older people in India. Future studies are demanded to explore the causal relationship and to reveal the underlying mechanism.

**Supplementary Information:**

The online version contains supplementary material available at 10.1186/s12889-024-19181-7.

## Introduction

As is well known, falls are age-related and common in older people, constituting a common and serious health issue for the elderly. With ageing, there is a decline in the body’s balance, coordination, and muscle strength, leading to an increased risk of falls. Besides, both social and psychological factors contribute to a higher prevalence in older adults [1]. According to the latest WHO data, approximately 28–35% of people aged 65 and older experienced falls each year globally. And this incidence increased to 32–42% among those aged 70 and older [2]. Actually, on account of limited medical resources, environmental factors, malnutrition and lack of health education and preventive measures, the elderly suffers higher risk of falls in developing countries [2–4]. Studies on falls in India have found that the incidence of falls was higher in rural populations compared to urban populations, which confirms that falls are a health event influenced by multiple factors [5]. The consequent adverse effects resulting from falls include hospitalization, disability even mortality [6]. Also, they bring about inevitable social and economic burdens globally [6, 7].

Nowadays, the ageing population is expanding worldwide, especially in developing countries like India. It leads to more health, economic and social challenges. India is projected to have the largest population globally by 2028, and dwellers aged 60 and above are projected to reach 19.5% total by 2050 [8]. Consequently, India is one of the countries facing the most severe ageing problems. And an increase in life expectancy does not mean a higher quality of life on account of chronic diseases, depression, disability or independence for self-care and other age-related disorders which older adults still confront. To provides a comprehensive framework for understanding and promoting the health and well-being of older adults, successful ageing was put forward. The concept of successful ageing was initially introduced in 1987, highlighting three key components: low probability of disease and related disability, high cognitive and physical functional capacity, and active engagement with life [9]. Over the years, the concept has evolved and expand, and combined physical and functional indicators with psychological and social factors [10]. Hence, exploring successful ageing and the factors affecting it will be crucial to assume the ageing process and promoting the quality of life of older adults.

Current researches on the impact of falls on older adults’ health primarily focused on negative consequence such as injuries and fractures [11, 12]. However, the relationship between falls themselves, including those that do not result in injuries, and overall health remains unclear. To investigate this, we explored the correlation between falls and successful ageing using the database of LASI and validated our findings across different subgroups of populations.

## Methods

### Study participants

Data used in the study were based on the wave one survey (2017-18) of the Longitudinal Ageing Study in India (LASI), a comprehensive national longitudinal survey of 72,250 adults. The database involves all regions of India and provides information on social, physical and psychological factors relevant to the ageing process in a large population [13]. The data collected in LASI wave 1 depended on the stratified area probability cluster sampling. Each participant was signed a written informed consent form, details of which can be found at https://lasi-india.org and https://g2aging.org [14].

**Fig. 1:**
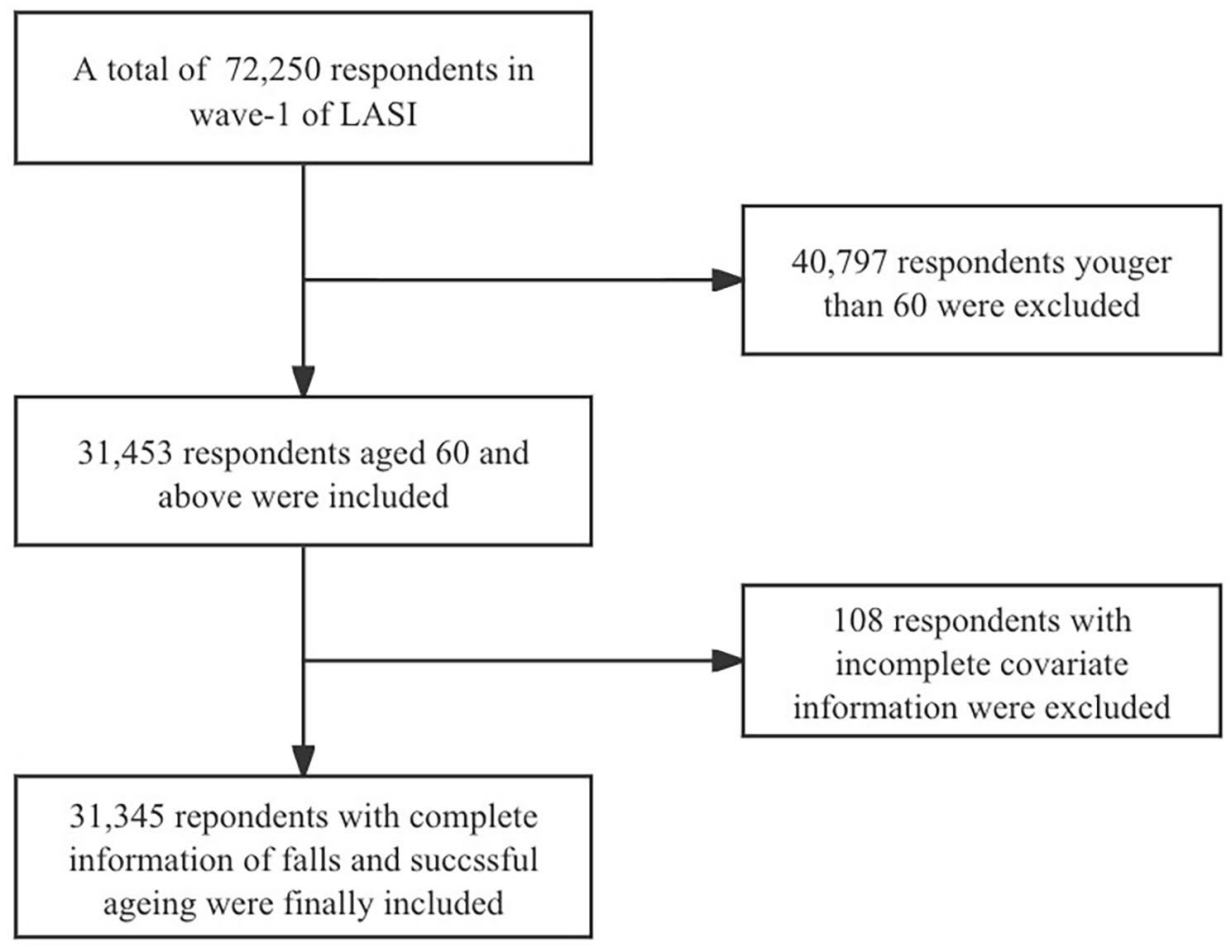
The flowchart of the study

Our present study restricted respondents aged 60 and above, which included 31,453 eligible participants. Then, the individuals with incomplete information on falls and successful ageing were excluded(*n* = 108). Finally, 31,345 participants enrolled in the present study. The detailed flowchart was shown in Fig. 1.

### Successful ageing assessment

The outcome variables were determined as successful ageing, of which there were no exact standards to define. Successful ageing was defined following the Rowe-Kahn’s model. Older adults with high cognitive function, active social engagement and without disability, chronic diseases nor depressive symptoms were classified into it. Details of it can be found in the appendix.

### Falls, fall-injury and multiple falls

The falls, fall-injury and multiple falls assessments depended on a face-to-face interview. The falls among participants were assessed by asking if they fell down in last two years. Participants who injured themselves and needed medical help in falls were classified into a fall-injury group. Respondents who reported twice or more falls were believed to have multiple falls.

### Covariates

Socio-demographic and biological variables were adjusted to render our results more reliable. Sociodemographic variables included age, sex, educated level, working status, marital status, place of residence, annual per capita consumption expenditure, caste and religion. Annual per capita consumption was assessed by household consumption data, which represented economic status based on former studies [15, 16]. Both food expenditure and non-food expenditure were collected by questionnaires to complete the data. Caste, India’s unique racial system, means the socioeconomic status of people in India [17].

Besides, the ageing process was associated with biological and behavioral factors based on the studies before [18]. Therefore, we included body mass index (BMI), smoking status, drinking status and physical activity status. Smoking status was assessed by consumption of tobacco in a lifetime, as well as drinking status. Physical activity status was assessed using moderate physical activities, including cleaning the house, washing clothes, fetching water or wood, drawing water from a well, gardening, bicycling at a regular pace, walking at a moderate pace, dancing, or floor or stretching exercises. And physical activity status was coded according to the frequency. Other specific categories of covariates were shown in the Table 1.

**Table 1:** Baseline characteristics of participants

Characteristics	Total	Falls		
		No (*n* = 24,998)	Yes (*n* = 6347)	*p*-value
**Age**	68.85 ± 7.47	68.72 ± 7.36	69.36 ± 7.85	< 0.001
**Sex, n(%)**				< 0.001
Man	15,030 (47.95%)	12,423 (49.70%)	2607 (41.07%)	
Woman	16,315 (52.05%)	12,575 (50.30%)	3740 (58.93%)	
**Level of education, n (%)**				< 0.001
Less than 5 years complete	22,652 (72.27%)	17,757 (71.03%)	4895 (77.12%)	
5–10 years complete	6079 (19.39%)	4999 (20.00%)	1080 (17.02%)	
10 more years complete	2614 (8.34%)	2242 (8.97%)	372 (5.86%)	
**BMI, kg/m2, n (%)**				< 0.001
< 18.5	6519 (23.26%)	5045 (22.50%)	1474 (26.31%)	
18.5–25	14,689 (52.42%)	11,859 (52.89%)	2830 (50.52%)	
25–30	5183 (18.50%)	4232 (18.88%)	951 (16.98%)	
≥ 30	1631 (5.82%)	1284 (5.73%)	347 (6.19%)	
**Marital status, n (%)**				< 0.001
Married or Partnered	20,063 (64.01%)	16,323 (65.30%)	3740 (58.93%)	
Widowed	10,637 (33.94%)	8144 (32.58%)	2493 (39.28%)	
Others	645 (2.06%)	531 (2.12%)	114 (1.80%)	
**Place of residence, n (%)**				< 0.001
Urban	10,688 (34.10%)	8849 (35.40%)	1839 (28.97%)	
Rural	20,657 (65.90%)	16,149 (64.60%)	4508 (71.03%)	
**Working status, n (%)**				0.014
Currently unemployed	20,636 (65.84%)	16,374 (65.50%)	4262 (67.15%)	
Currently employed	10,708 (34.16%)	8623 (34.50%)	2085 (32.85%)	
**Annual per capita consumption expenditure, n(%)**				0.421
Low	11,095 (35.40%)	8887 (35.55%)	2208 (34.79%)	
Middle	10,605 (33.84%)	8456 (33.83%)	2149 (33.86%)	
High	9643 (30.77%)	7653 (30.62%)	1990 (31.35%)	
**Physical activity status, n(%)**				0.002
Frequent	14,741 (47.28%)	11,664 (46.93%)	3077 (48.66%)	
Rare	4366 (14.00%)	3447 (13.87%)	919 (14.53%)	
Never	12,071 (38.72%)	9744 (39.20%)	2327 (36.80%)	
**Drinking status, n (%)**				0.015
Never	25,824 (82.83%)	20,663 (83.13%)	5161 (81.64%)	
Current	2756 (8.84%)	2169 (8.73%)	587 (9.29%)	
Ever	2598 (8.33%)	2024 (8.14%)	574 (9.08%)	
**Smoking status, n (%)**				< 0.001
Never	24,857 (79.74%)	19,711 (79.30%)	5146 (81.46%)	
Current	4368 (14.01%)	3581 (14.41%)	787 (12.46%)	
Ever	1948 (6.25%)	1564 (6.29%)	384 (6.08%)	
**Successful ageing, n(%)**				< 0.001
No	23,508 (75.00%)	18,358 (73.44%)	5150 (81.14%)	
Yes	7837 (25.00%)	6640 (26.56%)	1197 (18.86%)	
**Absence of chronic diseases, n(%)**				< 0.001
No	16,452 (52.54%)	12,839 (51.41%)	3613 (57.00%)	
Yes	14,860 (47.46%)	12,134 (48.59%)	2726 (43.00%)	
**Freedom from disability, n(%)**				< 0.001
No	6677 (21.33%)	4758 (19.06%)	1919 (30.26%)	
Yes	24,632 (78.67%)	20,210 (80.94%)	4422 (69.74%)	
**High cognitive ability, n(%)**				< 0.001
No	5354 (17.39%)	4147 (16.87%)	1207 (19.44%)	
Yes	25,439 (82.61%)	20,438 (83.13%)	5001 (80.56%)	
**Free from depressive symptoms, n(%)**				< 0.001
No	2331 (7.62%)	1565 (6.41%)	766 (12.40%)	
Yes	28,267 (92.38%)	22,855 (93.59%)	5412 (87.60%)	
**Active social engagement in life, n(%)**				< 0.001
No	6487 (20.94%)	5003 (20.26%)	1484 (23.58%)	
Yes	24,494 (79.06%)	19,685 (79.74%)	4809 (76.42%)	
**Caste, n(%)**				< 0.001
Scheduled caste	5123 (16.46%)	3932 (15.85%)	1191 (18.85%)	
Scheduled trible	5200 (16.71%)	4475 (18.04%)	725 (11.48%)	
Other backward class	11,830 (38.01%)	9315 (37.55%)	2515 (39.81%)	
No or other caste	8972 (28.83%)	7086 (28.56%)	1886 (29.86%)	
**Religion, n(%)**				< 0.001
Hindu	22,955 (73.24%)	18,056 (72.23%)	4899 (77.19%)	
Muslim	3714 (11.85%)	3017 (12.07%)	697 (10.98%)	
Christian	3136 (10.01%)	2766 (11.07%)	370 (5.83%)	
Others	1539 (4.91%)	1158 (4.63%)	381 (6.00%)	

SD, standard deviation; BMI, body mass index

Mean ± SD for continuous variables: *P* value was calculated by Kruskal Wallis H test;

Number (%) for categorical variables: *P* value was calculated by chi-square test

### Statistical analysis

Mean and standard deviation were presented to express continuous variables. Meanwhile, proportions were presented to describe categorical variables. The Kruskal Wallis H test and chi-square test were used to calculate inter-group statistical differences in baseline characteristics for continuous variables and categorical variables, respectively. Moreover, multivariate logistic regression was conducted to find the associations between falls, fall-injury, multiple falls and successful ageing with odds ratios (ORs) and 95% confidence intervals (CIs). For diverse adjust factors, we conducted two models. Sociodemographic covariates mentioned above were adjusted in model 1. Then we added biological covariates to adjust in model 2.

Moreover, we conducted interaction analyses in subgroups, including age, sex, drinking status, smoking status and place of residence. Three age groups were divided: <70, 70–79 and ≥ 70 years. Drinking status, smoking status and place of residence were categorized as mentioned above. Stratified linear regression models were performed in each subgroup. And in order to compare models with and without covariate interaction, the log-likelihood ratio test was used.

The statistical software packages R (http://www.R-project.org, The R Foundation) and Empower (http://www.empowerstats. com) were used to conduct all statistical analyses. And the results were regarded to be statically significant if a *P*-value < 0.05.

## Results

### Baseline characteristics

31,345 participants total, whose mean age was 68.85, were included in the current study. Table 1 showed their characteristics and covariates. Among all subjects, the prevalence of falls was 20.25%, and they were more likely to be older, women, lower level of education, BMI < 18.5 or ≥ 30, widowed, living in a rural area, currently unemployed, higher consumption expenditure, more frequent physical activity, drinker and non-smoker. Besides, for successful ageing accession, people who fall tended to have higher incidences of chronic diseases, disability, depressive symptoms and lower cognitive ability and social engagement in life.

There 25% of participants in present study reported successful ageing. And 47.46%, 78.67%, 82.61%, 92.38% and 79.06% respondents who were absence of chronic diseases, free from disability, high cognitive ability, free from depressive symptoms and active social engagement were reported, respectively. 18.86% of participants with falls reported successful ageing compared 26.56% without falls.

### Falls and associated successful ageing

Table 2. represented the prevalence and the multivariable logistic regression estimates of falls, fall-injury, multiple falls and successful ageing in Indian adults aged 65 and above. After adjusting for sociodemographic covariates (model 1), successful ageing was negatively associated with falls for related respondents (OR 0.69; 95%CI 0.65–0.75). Then, after further adjusting for health characteristics (model 2), the odds of successful ageing among fall people remained significantly higher about the odds of successful ageing among non-fall people (OR 0.70; 95%CI 0.65–0.76). Similarly, successful ageing was significantly related to multiple falls both in model 1 (OR 0.68; 95%CI 0.61–0.75) and model 2 (OR 0.70; 95%CI 0.63–0.78). Inversely, the statistical result of successful ageing and fall-related injury did not show significance both in model 1 (OR 0.84; 95%CI 0.71-1.00) and model 2 (OR 0.86; 95%CI 0.72–1.04).

**Table 2:** Relationship between falls and successful ageing

		Unadjusted Model	Model 1	Model 2
	n	OR (95%CI)	OR (95%CI)	OR (95%CI)
Falls				
No	24,998	Ref.	Ref.	Ref.
Yes	6347	0.64 (0.60, 0.69)	0.69 (0.65, 0.75)	0.70 (0.65, 0.76)
Fall-injury				
No	1900	Ref.	Ref.	Ref.
Yes	4447	0.80 (0.68, 0.95)	0.84 (0.71, 1.00)	0.86 (0.72, 1.04)
Multiple falls				
< 2	3581	Ref.	Ref.	Ref.
≥ 2	2756	0.61 (0.55, 0.67)	0.68 (0.61, 0.75)	0.70 (0.63, 0.78)

OR odds ratio, 95% CI 95% Confidence interval;

Unadjusted model: no covariates were adjusted;

Model 1 adjust for: age, sex, level of education, marital status, place of residence, working status, caste, religion, annual per capita consumption expenditure;

Model 2 adjust for: age, sex, level of education, marital status, place of residence, working status, caste, religion, annual per capita consumption expenditure, physical activity status, BMI, drinking status; smoking status

### Subgroup analysis

**Fig. 2:**
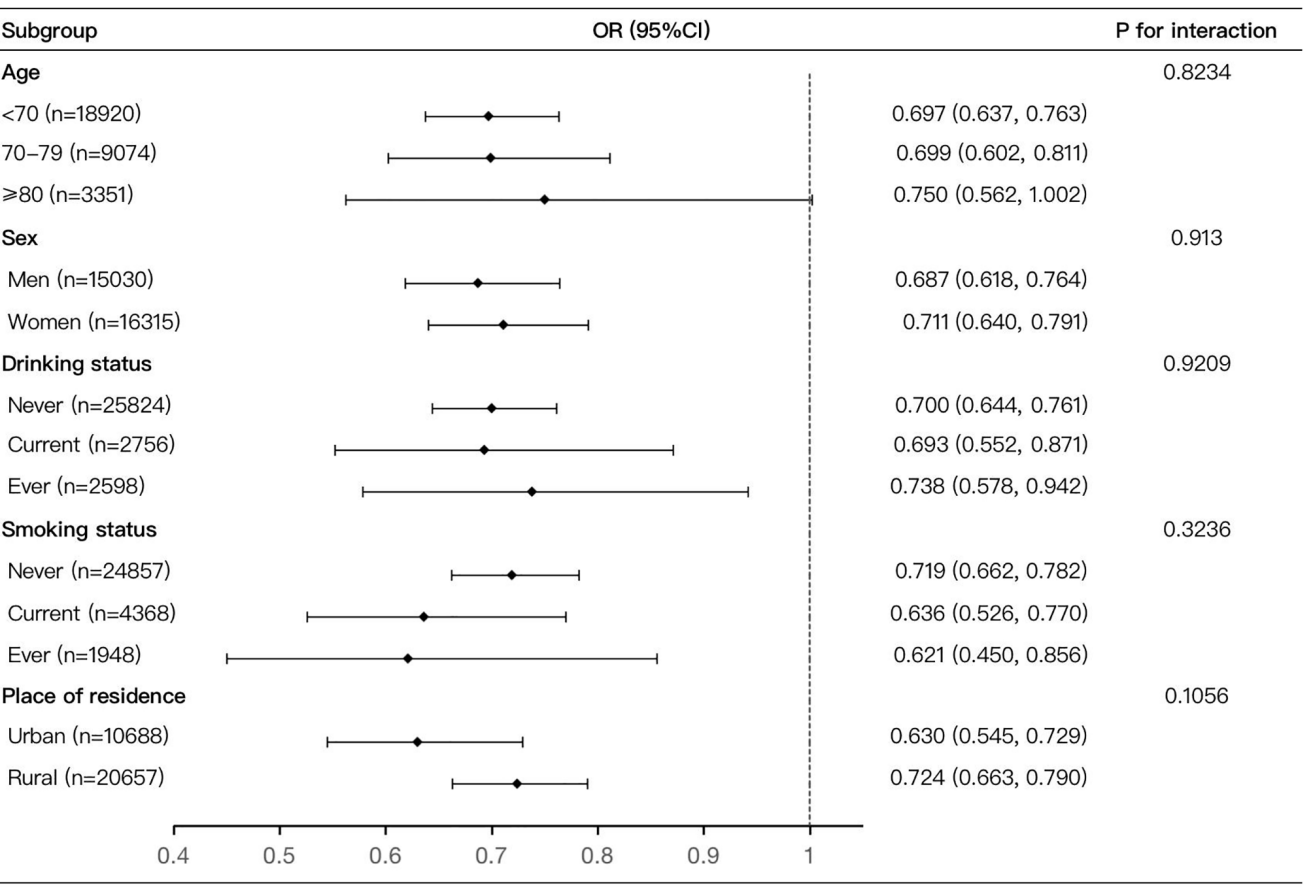
Subgroup analysis of relationship between falls and successful ageing. Age was recategorized into three groups: <70, 70–79, ≥ 80 years. OR, odds ratio; 95% CI, 95% Confidence interval. Model 2 adjusted for: age, level of education, work status, marital status, religion, place of residence, living arrangement, economic status, caste, body mass index (BMI), vigorous physical activity, waist-to-hip ratio, number of chronic diseases, self-rated health (SRH), drinking status, smoking status, depression, pain

To further explore the association between falls and successful ageing, we conducted subgroup analysis and interaction tests presented in Fig. 2., according to subgroup factors, including age, sex, drinking status, smoking status and place of residence. Besides, the stratified analyses by subgroup factors for the association between falls and successful ageing showed no significant interactions (*P* = 0.823, 0.913, 0.921, 0.324, 0.106, respectively). Notably, all the results of hierarchical analysis demonstrated that the association between falls and successful ageing was statistically significant.

## Discussion

Our study observed a negative correlation between falls and successful ageing regardless of injury in older people in India. Among all study samples, we found that 20.2% of older adults reported a history of falls, compared with 8.8% who reported multiple falls histories and 14.2% with fall-related injuries. The prevalence of successful ageing in our study was 25.0%, with an incidence of 26.6% among non-fallers and 18.86% among seniors who fell.

In fact, we found a negative association between falls and successful ageing (OR 0.70; 95%CI 0.65–0.76). However, this association disappeared among older adults whose falls did not result in injuries (OR 0.86; 95%CI 0.72–1.04), while it persisted among those who experienced multiple falls (OR 0.70; 95%CI 0.63–0.78). The results demonstrated that the association between successful ageing and fall-related injury didn’t show a statistical significance, which proved that the association was established regardless of injury in the elderly in India. It suggests that the attention should not be solely focused on the adverse events caused by falls, but that falls themselves indicate unfavorable ageing events.

Indeed, physiological factors including age, gender, physical activity status, BMI, alcohol consumption, and smoking status may influence the connecting of falls and successful ageing. On one hand, individuals who are older, female (especially postmenopausal women), outside the normal range of BMI (either higher or lower), less active, consume more alcohol, or smoke more are more prone to falls. That could be attributed to decreased muscle mass or function, inadequate balance and coordination, lower bone density, or an increased risk of chronic diseases in these population groups [19–23]. On the other hand, these physiological factors can also have a negative impact on successful ageing. The process of ageing involves a decrease in both physical and cognitive abilities as well as social engagement, which leads to a lower level of ageing life. A large-scale cohort study found that individuals aged 70 and above experience significantly more severe and longer depressive symptoms compared to younger individuals [24]. Compared to males, females often face a higher incidence of depression due to hormonal fluctuation and psychosocial factors [25, 26]. Moreover, elderly women demonstrate a heightened probability of cognitive impairments, encompassing Alzheimer’s disease and other forms of dementia [27, 28]. And the impact of BMI, physical activity, smoking, and alcohol consumption on the quality of life in older adults is multifaceted. Both high and low BMI, lower level of physical activity, as well as smoking and excessive alcohol consumption, may be associated with increased risks of chronic diseases and cognitive impairments [29–33].

Some social demographic factors indeed serve as important factors in the correlation between falls and successful ageing. In fact, economic status, educational level, and the unique caste system and religion in India all influence an individual’s socioeconomic status. And lower socioeconomic status predicted a higher falls rate [34, 35]. Older adults with lower socioeconomic status may have less health knowledge and fewer health-promoting behaviors, and they may also reside in less safe environments [36]. Additionally, economic difficulties can limit their access to medical services, including preventive measures and rehabilitation programs, thereby increasing the risk of falls. Researches have shown that individuals with lower socioeconomic status are at higher risk for a range of chronic diseases, such as coronary artery disease, type 2 diabetes, and cancers, as well as premature mortality [37, 38]. Numerous studies have documented the effects of socioeconomic status on age-related declines in physical and cognitive functions [39, 40]. Furthermore, individuals with lower socioeconomic status are less likely to be classified as ageing successful [41–43].

Overall, the decline in physical function, cognitive abilities, balance, and the presence of depression during the aging process may simultaneously contribute to falls and successful ageing. Moreover, we can consider that fractures, pain, and even disability resulting from falls act as the bridge linking falls to successful ageing. The adverse outcomes of falls can affect various dimensions of an older adult’s life, including physical, psychological, and social factors, thereby detrimentally impacting their quality of life and successful ageing [6, 7]. Conversely, older adults with chronic diseases, mental health disorders, and low levels of social engagement—indicators of unsuccessful ageing—may exhibit an increased risk of falls [44–47]. However, in our study, fall related injuries did not show the association with successful ageing. It indicated that our study underscored the significance of fall prevention in older adults as a critical measure, rather than merely preventing injuries after falls. The occurrence of falls, irrespective of injuries, signified adverse progression in the ageing process.

Moreover, we adopted Rowe and Kahn’s model to define successful ageing. Compared to other studies, we include a broader range of chronic diseases. Besides the more commonly included conditions such as cancer, chronic lung disease, diabetes, heart disease, and stroke, our definition also encompasses bone or joint disease, neurological or psychiatric disease, and high cholesterol [33, 48, 49]. For cognitive assessment, we evaluated participants’ cognitive abilities across multiple domains, including memory, orientation, arithmetic function, executive function, and object naming. Participants were scored, and those within the lowest 10th percentile were classified as the low cognitive group. It allowed for a more comprehensive understanding of older individual’s overall health status, capturing a wider spectrum of potential health issues that may impact ageing. Also it enabled the identification of less commonly considered but significant conditions that contribute to the aging process, thereby providing a more nuanced and accurate assessment of successful ageing.

Our study had several strengths. The large size of data collected from different regions and socio-economic backgrounds all around the country ensured a high quality of representativeness and universality of our research. To ensure the reliability of our results, our data analyses were adjusted for multiple variables, from biological to sociodemographic. Besides, we applied a diverse concept to assess successful ageing, including effective assessment of cognitive ability and depressive symptoms and objective measures of functions. And to the best of our knowledge, this was the first research demonstrating a linkage between falls and successful ageing.

However, we certainly had some limitations that were supposed to be acknowledged. First, based on the cross-sectional design, the results concluded were not causal but only associated. But future waves of LASI can support follow-up studies of potential causal relationships. Second, chronic diseases and other health conditions were dependent on self-report, which mean recall bias or the several levels to understand questions might influence the rate of successful ageing. Third, the lack of universal methods of reporting falls and the time limitation of falls history recall could prevent elderly individuals from giving correct information. Thus, the prevalence of falls could be underestimated. Finally, the subjective character of socio-demographics and the limitation of potential co-variates could entail multiple interpretations of our conclusion.

## Conclusions

Falls, multiple falls are negatively associated with successful ageing, regardless of covariates. While fall-related injuries don’t show a statically significant association with successful ageing. Owing to the emerging ageing population, public concerns put on older adults and ageing process. Future researches are needed to explore deep relationship and reveal the underlying mechanism.

### Electronic supplementary material

Below is the link to the electronic supplementary material.

Supplementary Material 1

## Data Availability

The data presented and analysed in the study are available at https://lasi-india.org.

## Abbreviations

LASI: Longitudinal Ageing Study in India
BMI: Body mass index
OR: Odds ratio
95% CI: 95% Confidence interval
