# Supplementary material for: Successful ageing is associated with falls among older adults in India: a large population based across-sectional study based on LASI
Source: BMC Public Health. 2024 Jun 24;24:1682. doi: 10.1186/s12889-024-19181-7 (PMC11197243; doi:10.1186/s12889-024-19181-7)
Supplement: Supplementary file 1 — Supplementary Material 1 [file 12889_2024_19181_MOESM1_ESM.docx]

**Appendix**

The definition of successful ageing and the components in detail were as follow:

1. Absence of chronic diseases: Participants were received the question “Have you been diagnosed with conditions listed below by a doctor?” to assess chronic diseases. The diseases relevant included hypertension, chronic heart diseases, stroke, chronic lung disease, diabetes, cancer or malignant tumor, bone or joint disease, neurological or psychiatric disease and high cholesterol[1]. Respondents were considered having no chronic diseases if they reported none of these above.
2. Freedom from disability: Respondents were classified as freedom from disability if they had no difficulty in performing any Active of Daily Living (ADL) independently. ADL is used to refer to normal daily self-care activities, including movement in bed, changing position from sitting to standing, feeding, bathing, dressing, grooming and personal hygiene. Whether people can perform ADLs independently without difficulty was to measure individuals’ functional status, especially in older adults or people with disability[2].
3. High cognitive ability: Cognitive function in the LASI survey was measured through memory, orientation, arithmetic function, executive function and object naming adapted from the Mini-Mental State Examination (MMSE)[3], as well as the cognitive module of the United States Health and Retirement Study (HRS)[4], China Health and Retirement Longitudinal Study (CHARLS), and the Mexican Health and Aging Study (MHAS)[5]. Immediate words recall and delayed words recall were used to measure memory. Time and place measures were used to assess orientation. Backward counting, a serial seven subtraction task which involved two computations, was used to measure arithmetic function[4]. Besides, paper folding, pentagon drawing and object naming methods were also used to further measure the cognitive functions among older adults[5]. A composite score of 0–43 was computed using the domain wise measures. The lowest 10^th^ percentile was defined as poor cognitive ability. Respondents except whose score in lowest 10^th^ percentile were classified in high cognitive group[6].
4. Free from depressive symptoms: The symptoms of probable depression among older adults were assessed using the Short Form Composite International Diagnostic Interview (CIDI-SF) with the final score of 3 or more indicating major depressive disorder (MDD)[7, 8]. Respondents who didn’t meet the standards of “MDD” were defined as “free from depressive symptoms”.
5. Active social engagement: Respondents were said to be socially engaged if they participated in the following activities: eat out of the house (restaurant/ hotel); go to park/ beach for relaxing/ entertainment; play cards or indoor games; play outdoor games/ sports/ exercise/ jog/ yoga; visit relatives/ friends; attend cultural performances/ shows/ cinema; attend religious functions/ events such as bhajan/ Satsang/ prayer; attend political/ community/ organization group meetings; read books/ newspapers/ magazines; watch television/ listen to the radio and use a computer for e-mail/ net surfing.

**Reference List**

1. 1. Luo, H., et al., *Association between obesity status and successful aging among older people in China: evidence from CHARLS.* BMC Public Health, 2020. **20**(1): p. 767.
2. 2. Srivastava, S. and T. Muhammad, *Violence and associated health outcomes among older adults in India: A gendered perspective.* SSM Popul Health, 2020. **12**: p. 100702.
3. 3. Juva, K., et al., *Functional assessment scales in detecting dementia.* Age Ageing, 1997. **26**(5): p. 393-400.
4. 4. Blankson, A.N. and J.J. McArdle, *A Brief Report on the Factor Structure of the Cognitive Measures in the HRS/AHEAD Studies.* J Aging Res, 2014. **2014**: p. 798514.
5. 5. Saenz, J.L., et al., *Household use of polluting cooking fuels and late-life cognitive function: A harmonized analysis of India, Mexico, and China.* Environ Int, 2021. **156**: p. 106722.
6. 6. Pandav, R., et al., *Sensitivity and specificity of cognitive and functional screening instruments for dementia: the Indo-U.S. Dementia Epidemiology Study.* J Am Geriatr Soc, 2002. **50**(3): p. 554-61.
7. 7. Muhammad, T., T. Meher, and T.V. Sekher, *Association of elder abuse, crime victimhood and perceived neighbourhood safety with major depression among older adults in India: a cross-sectional study using data from the LASI baseline survey (2017-2018).* BMJ Open, 2021. **11**(12): p. e055625.
8. 8. Muhammad, T. and T. Meher, *Association of late-life depression with cognitive impairment: evidence from a cross-sectional study among older adults in India.* BMC Geriatr, 2021. **21**(1): p. 364.
